# Metatranscriptomic Analysis of Multiple Environmental Stresses Identifies *RAP2*.4 Gene Associated with *Arabidopsis* Immunity to *Botrytis cinerea*

**DOI:** 10.1038/s41598-019-53694-1

**Published:** 2019-11-18

**Authors:** Arjun Sham, Hibatullah Al-Ashram, Kenna Whitley, Rabah Iratni, Khaled A. El-Tarabily, Synan F. AbuQamar

**Affiliations:** 1 Department of Biology, United Arab Emirates University, 15551 Al-Ain, UAE; 2 School of Veterinary and Life Sciences, Murdoch University, Murdoch, Western Australia 6150 Australia

**Keywords:** Plant stress responses, Biotic

## Abstract

In this study, we aimed to identify common genetic components during stress response responsible for crosstalk among stresses, and to determine the role of differentially expressed genes in *Arabidopsis*-*Botrytis cinerea* interaction. Of 1,554 *B. cinerea* up-regulated genes, 24%, 1.4% and 14% were induced by biotic, abiotic and hormonal treatments, respectively. About 18%, 2.5% and 22% of *B. cinerea* down-regulated genes were also repressed by the same stress groups. Our transcriptomic analysis indicates that plant responses to all tested stresses can be mediated by commonly regulated genes; and protein-protein interaction network confirms the cross-interaction between proteins regulated by these genes. Upon challenges to individual or multiple stress(es), accumulation of signaling molecules (e.g. hormones) plays a major role in the activation of downstream defense responses. *In silico* gene analyses enabled us to assess the involvement of RAP2.4 (related to AP2.4) in plant immunity. *Arabidopsis RAP2.4* was repressed by *B. cinerea*, and its mutants enhanced resistance to the same pathogen. To the best of our knowledge, this is the first report demonstrating the role of *RAP2.4* in plant defense against *B. cinerea*. This research can provide a basis for breeding programs to increase tolerance and improve yield performance in crops.

## Introduction

Plants frequently have to cope with a wide array of environmental (abiotic and biotic) challenges. In nature, simultaneous or sequential exposure of plants to multiple stress conditions *i.e*., more than one abiotic and/or biotic stress, occurs more often than to a single individual stress^1,2^. Plants have evolutionarily developed sophisticated adaptation and defense mechanisms^3^. Thus, this response cannot be predicted based on a plant response to an individual stress. Depending on the length, intensity and severity of stresses, the complexity of plant response can be orchestrated by the integration of a number of metabolic pathways and the crosstalk between different signal transduction pathways^4^. The specificity of response can be determined by an array of mechanisms, which may crosstalk or diverge, and form complex networks including transcription factors (TFs), kinases and reactive oxygen species that may interact with each other^3^. As a result, plants tune gene expression along with their physiological needs to promote adaptation to short- and long-term environmental changes.

There are a number of global transcriptome analyses investigating various environmental stresses in plants including *Arabidopsis thaliana*. Although many microarray studies have focused on individual stresses^5,6^, there is growing evidence that a unique gene expression can be activated in plants under simultaneous abiotic and/or biotic challenges. Researchers have identified specific and common molecular responses to bacterial, fungal and viral pathogen attacks; others have analyzed plant responses to multiple abiotic stresses using different transcriptomic tools^7–9^. In their efforts to compare between different stress responses, two microarray studies identified the genes and pathways that are commonly induced by the necrotrophic fungal pathogen *Botrytis cinerea* (biotic) and abiotic (salt, heat, cold, drought, osmotic and oxidative stress) threats^10,11^. The effects of simultaneous biotic and abiotic stresses may interact either synergically or antagonistically. Hence, genes and molecular mechanisms involved in regulating plant responses are mainly associated with signal molecules known as plant hormones^1,12,13^.

Several studies have reported that the major phytohormones, salicylic acid (SA), jasmonates (JA), ethylene (ET) and abscisic acid (ABA), are involved in the regulation of plant response to the adverse effect of biotic and abiotic stresses^14,15^. To lesser extent, other hormones such as auxins, gibberellic acid, cytokinins, brassinosteroids and strigolactones may also play a role in plant defense signalling pathways^14,16,17^. Studies have reported the association of different defense signaling pathways in response to pathogens and described that among the co-expressed genes, a majority is co-induced or -repressed with the treatment of SA and methyl-JA (MeJA)^9,17^. Plant response to certain insects can also be related to hormone signalling. For example, a response of wounding by insects such as *Spodoptera exigua* (Hübner) and *Pieris rapae*, the level of JA, JA-isoleucine and ET was increased^18^. In tomato (*Solanum lycopersicum*), *tomato protein kinase* 1*b* (*TPK1b*) RNA interference (RNAi) mutant plants showed increased susceptibility to *B. cinerea* and the herbivorous insect *Manduca sexta*, and increased sensitivity to 1-aminocyclopropane-1-carboxylic acid (ACC), the natural precursor of ET^19^. In addition, ectopic expression of *TPK1b* in *Arabidopsis* conferred increased resistance to *B. cinerea* and *Alternaria brassicicola*. This suggests that TPK1b and ET as key regulators of insect and pathogen defense responses.

*B. cinerea* is considered the second most important plant pathogen, causing significant economic damage on over 200 crops worldwide^20^. Moreover, *B. cinerea* has become an important model for studying interactions between plants and necrotrophic pathogens^21^. Typically, SA, JA and ET play major roles in response to pathogen infections; while ABA is responsible for plant defense against abiotic stresses^15,22^. Hence, the existence of crosstalk in plant responses to biotic and abiotic stresses involves various signaling hormone pathways. For instance, ET and JA have been found to be associated with elevated expression levels of the plant defense gene *PDF1.2*, which contributes to resistance to *B. cinerea* and *A. brassicicola*^23^. *Arabidopsis* and tomato mutants with altered ET and JA signaling pathways are susceptible to *B. cinerea* infection^5,24^. Exogeneous treatment of *Arabidopsis* with ET or MeJA substantially decreased *B. cinerea* infection, indicating the crucial function of ET and JA in *B. cinerea* resistance. Furthermore, a study has confirmed the role of *expansin-like A2* (*EXLA2*) gene in defense against *B. cinerea* and tolerance to abiotic stresses^16^. In *Arabidopsis*, the negative regulation of *WRKY57* against *B. cinerea* is dependent on the JA signaling pathway^25^. Another gene from the WRKY family, *WRKY33* is found to be important in *B. cinerea* resistance^26,27^. Molecular and genetic studies reveal that ABA negatively influences defense to *B. cinerea* and affects JA/ET and SA levels. Susceptibility/resistance can be determined by the antagonistic effect of ABA on JA, and this crosstalk requires suppression of *WRKY33* at early infection stages^28^. This indicates that *B. cinerea* promotes disease by suppressing WRKY33-mediated host defense.

It is important to study plant responses to multiple stress conditions in order to identify commonly regulated genes and pathways altered by these stresses. This will help to facilitate specific target gene manipulation for genetic engineering to enhance multiple stress tolerance in plants. There are several gene expression experiments as well as datasets in publicly available data repositories. Meta-analyses of public datasets can yield more biologically and technically sound information than that of individually analyzed datasets^29^. In addition, results coming from single stress experiments may be inaccurate or skewed if the compared datasets are not genetically equivalent, which is in contrast to analyzing public consistent datasets. The dynamics of whole‐transcriptome profiles of *Arabidopsis* exposed to sequential double stresses treated with combinations of *B. cinerea*, *P. rapae* and drought stress has been analyzed^12^. The study revealed that around one-third of the differentially expressed genes (DEGs) were shared by at least two single stresses.

In the present study, we analyzed and compared the public transcriptomic data to identify the overlapping stress-regulated genes in response to *B. cinerea* and other biotic (*Pseudomonas syringae* pv. *tomato DC3000* virulent and avirulent *Rpm1* strains*, A. brassicicola* and *P. rapae*), abiotic (oxidative stress and wounding) and hormonal (SA, JA, ET and ABA) stresses. These data were further analyzed to sort the up- and down-regulated genes in all stresses. This study revealed the genes uniquely expressed in response to each of these stresses, and those commonly expressed in response to *B. cinerea* and other stresses. The *Arabidopsis* Protein-protein Interaction (PPI) network demonstrated the complex regulatory network co-expressed across the multiple stresses. We also developed a transcriptome-interactome mapping strategy to compare the interactions between proteins encoded by genes that belong to different expression-profiling clusters. T-DNA insertion mutant lines of *related to AP2.4d* (*RAP2.4d*, *At1g22190*; further referred to as *RAP2.4*) exhibited increased resistance to *B. cinerea*, with reduced disease symptoms and pathogen growth in inoculated plants. Thus, a better understanding of the molecular mechanisms of plant responses to *B. cinerea* and other environmental stresses along with their genetic control has been developed. This will accelerate the future of genetic engineering and breeding programs for the production of crops with less of chemical pesticides.

## Results

### Screening of DEGs in response to individual stresses

**Figure 1:**
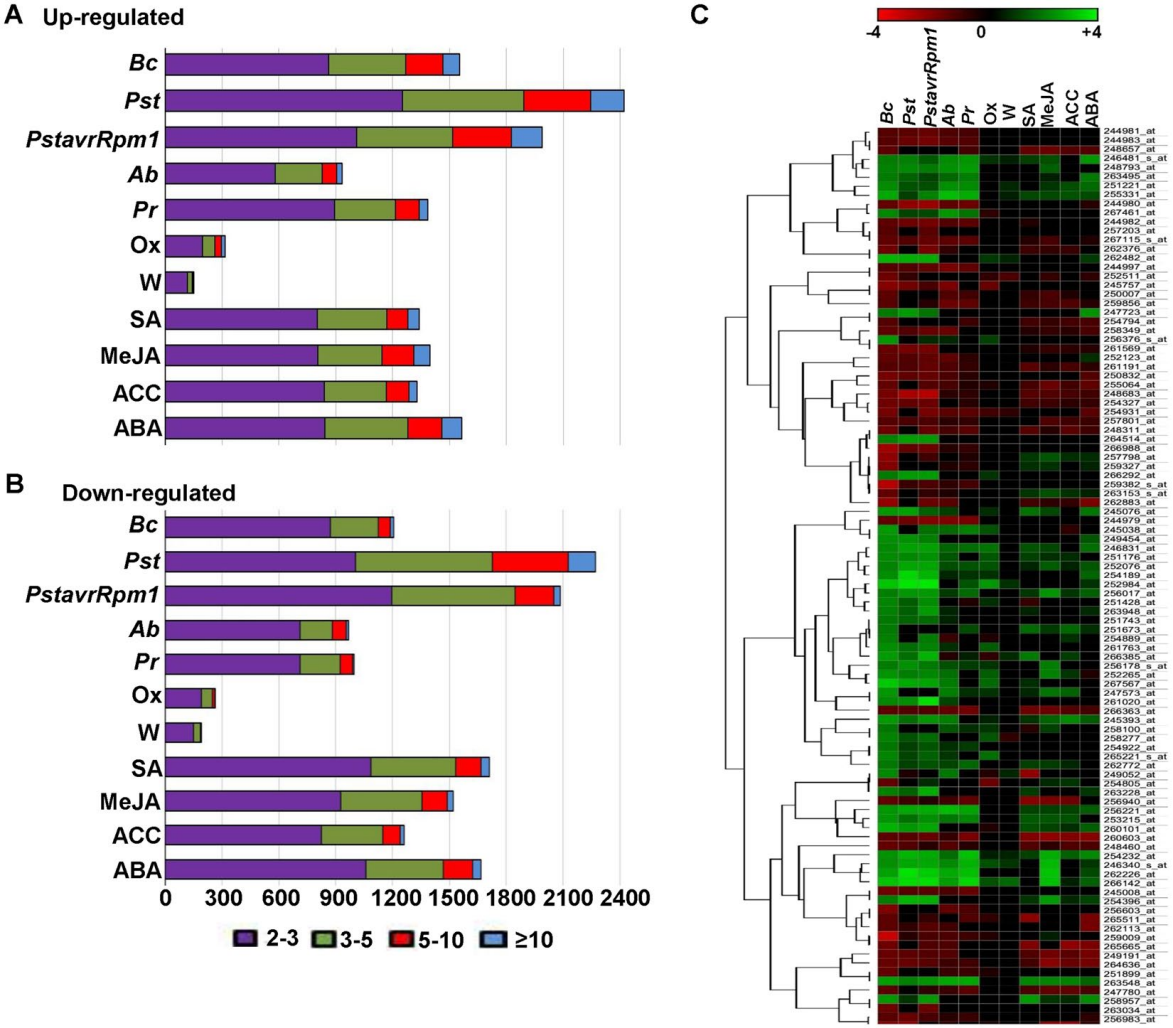
Gene expression analysis in *Arabidopsis* plants in response to different stress treatments. The number of (**A**) up- and (**B**) down-regulated genes in response to individual biotic, abiotic and hormonal stresses were plotted. (**C**) Top 50 up- and down-regulated genes in each individual stress were sorted and their expression profile in log_4_ values were plotted using a heatmap and linked to their relationship with dendrogram. *Bc*, *Botrytis cinerea*; *Pst*, *Pseudomonas syringae* pv. *tomato DC3000*; *PstavrRpm1*, *P. syringae* pv. *tomato avrRpm1*; *Ab*, *Alternaria brassicicola*, *Rp*, *Peris rapae*; Ox, oxidative stress; W, wounding; SA, salicylic acid; MeJA, methyl-jasmonate; ACC, 1-aminoacyclopropane-1-carboxylate; ABA, abscisic acid.

We aimed to identify unique and common DEGs among transcriptomic datasets related to environmental and hormonal stresses in *Arabidopsis*. Similar to previous studies^5,10,11^, *B. cinerea* up-regulated genes (*BUG*s) and *B. cinerea* down-regulated genes (*BDG*s) were identified based on their transcriptional levels in response to *B*. *cinerea* at 18 hours post inoculation (hpi). Using publicly available databases^30^, DEGs were also detected in *Arabidopsis* plants infected with *B. cinerea* at 18 hpi (Dataset S1, Supporting Information). For each individual dataset, the complete list of pathogen/pest infection (*P. syringae* pv. *tomato DC3000* and *avrRpm1*, *A. brassicicola* or *P. rapae*), abiotic stress (wounding and oxidative stress) or hormone treatment (SA, MeJA, ACC and ABA) (Fig. S1–S2, Supporting Information) can be found in Datasets S2–S4 (Supporting Information). Microarray analysis showed 1554 *BUG*s (6.8% of *Arabidopsis* genome) and 1206 *BDG*s (5.3%) (Fig. 1A,B). In response to *P. syringae* pv. *tomato DC3000* and *avrRpm1*, we found 2422 and 1989 genes considered up-regulated, and 2270 and 2085 considered down-regulated, respectively. There were 1902 genes (933 up-regulated; 969 down-regulated) after inoculation with *A. brassicicola*, 2382 genes (1386 up- and 996 down-regulated) were detected as DEGs in *Arabidopsis* plants infested with *P. rapae*.

The least number of DEGs was observed in plants under the two treatments that belong to oxidative stress and wounding (Fig. 1A,B). This could be attributed to the natural adaptation of *Arabidopsis* to the abiotic stress group in comparison to other stress conditions^10,11^. In *Arabidopsis*, the gene expression levels upon individual treatments of SA, MeJA, ACC and ABA were altered for 3,051, 2,918, 2,590 and 3,231 transcripts, respectively, from which 1,340 (43.9%), 1,397 (47.9%), 1,328 (51.3%) and 1,564 (48.4%) genes were stress-induced genes. On average, most FC of the DEGs in all stresses ranged between two-fold or three-fold. Interestingly, we noticed that some genes were induced >10 fold or repressed <10 fold. Although each dataset may have its unique DEGs, this does not rule out the possibility that common genes can be identified across the categories of stresses.

To visualize the gene expression data of *B. cinerea* and other examined stresses, a heatmap of the top 50 *BUG*s and *BDG*s was generated (Fig. 1C). The heatmap was combined with clustering methods, displaying a group of genes/samples together based on the similarity of their gene expression pattern. Together, this can be the first step for identifying genes that are commonly regulated or biological signatures that are associated with multiple conditions.

### Highly conserved expression of common DEGs to multiple stress responses

**Figure 2:**
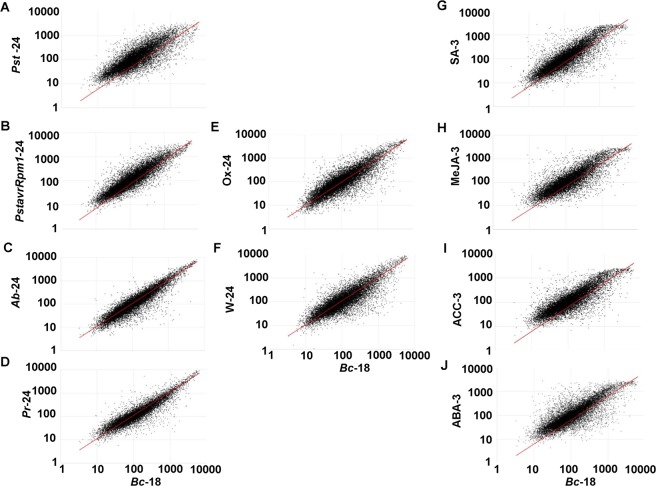
Comparison of gene expression in plants infected with *Botrytis cinerea* vs. challenged with environmental and hormonal stresses. Normalized expression value for each probeset in wild-type plants infected with *B. cinerea* at 18 hpi (*Bc*−18) is plotted on X-axis versus the expression value in wild-type plants after treatment with biotic, (**A**) *Pseudomonas syringae* pv. *tomato DC3000* (*Pst*-24), (**B**) *P. syringae* pv. *tomato avrRpm1*(*PstavrRpm1*-24), (**C**) *Alternraia brassicicola* (*Ab*-24), and (**D**) *Peris rapae* (*Pr*-24); abiotic, (**E**) oxidative stress (Ox-24), and (**F**) wounding (W-24); and hormonal (**G**) salicylic acid (SA-3), (**H**) methyl-jasmonate (MeJA-3), (**I**) 1-aminoacyclopropane-1-carboxylate (ACC-3), and (**J**) abscisic acid (ABA-3) stresses. hpi, hours post inoculation.

A scatter plot was constructed to compare the transcript level of the DEGs of each dataset with that altered by *B. cinerea* infection (Fig. 2). Clearly, our results demonstrated similar patterns of gene expression levels between *Arabidopsis* plants infected with *B*. *cinerea* and any individual stress at the time of treatment. This suggests that the mode of action of some DEGs common to multiple stresses may contribute in functions or processes that are common among responses to stress acclimation. We also determined the number (and percentage) of genes that were co-regulated in response to *B. cinerea* and single treatments of each stress type. In response to both the virulent and avirulent strains of *P. syrinage*, more than 2/3 and 1/2 of the genes were also induced and repressed, respectively, to *B. cinerea* (Table 1). Upon inoculation with *B. cinerea*, 40–50% of the genes were expressed by either the fungal pathogen *A. brassicicola* or the herbivory insect *P. rapae*. We also noticed that between 443 (28.5% in ACC) and 562 (36.2% in ABA) of the induced, and 429 (35.6% in ACC) and 532 (44.1% in SA) of the repressed genes in response to single hormonal stresses were co-regulated with infections by *B. cinerea*. To lesser extent, 193 (12.4%) and 69 (4.4%) of *BUG*s and 65 (5.4%) and 47 (3.9%) of *BDG*s were also up- and down-regulated by paraquat (oxidative stress) and wounding treatments, respectively. Only one gene was induced, and three genes were repressed in response to all individual stresses (Table 1). The list of identified DEGs that were co-regulated by *B. cinerea* and the rest of single stresses can be found in Datasets S5–S7 (Supporting Information).

**Table 1:** Common regulation of *Botrytis cinerea*-regulated genes with different treatments.

Treatment	Co-up-regulated genes		Co-down-regulated genes	
	Genes (count)	Percentage	Genes (count)	Percentage
*Pseudomonas syringae* pv. *DC3000*	1047	67.4	637	52.8
*P. syringae* pv. *avrRpm1*	1095	70.5	751	62.3
*Alternria brassicicola*	635	40.9	491	40.7
*Peris rapae*	761	49.0	548	45.4
Oxidative stress	193	12.4	65	5.4
Wounding	69	4.4	47	3.9
Salicylic acid	528	34.0	532	44.1
Methyl-jasmonate	510	32.8	478	39.6
1-aminoacyclopropane-1-carboxylate	443	28.5	429	35.6
Abscisic acid	562	36.2	511	42.4
All	1	0.1	3	0.2

Percentages of *B. cinerea* up-regulated genes (*BUG*s) and *B. cinerea* down-regulated genes (*BDG*s) that were also at least twofold increased or decreased by the biotic, abiotic and hormonal stresses.

Percentage = (Count of commonly up- or down-regulated genes of the treatment/count of *BUG*s (1554 genes) or *BDG*s (1206 genes)) *100%.

### Common DEGs and gene ontology (GO) functional enrichment analyses

Venn diagrams detected large overlaps in gene expression among the stress response treatments (Fig. S3, Supporting Information). By comparing *B. cinerea*-inoculated and biotic-stressed group, there were 380 and 218 commonly up- and down-regulated genes, respectively. Similarly, 211 genes were induced by *B. cinerea* infection as well as by all hormonal treatments, and 265 were repressed by the same group of treatments. Comparing *B. cinerea*-inoculated and abiotic stress-treated group, there were 21 commonly up-regulated genes and 30 commonly down-regulated genes (Fig. S3, Supporting Information). The overlapping DEGs in response to *B. cinerea* infection with all other stress categories are also listed (Dataset S8, Supporting Information). Excluding the repeated genes, the most significant 30 common DEGs (15 from each of up- and down-regulated genes) after combining biotic, abiotic and/or hormonal stress results with *BUG*s and *BDG*s were also determined (Table 2).

**Table 2:** Changes in expression of top 15 up- and down-regulated genes encoding putative proteins during *Botrytis cinerea* infection and/or other biotic, abiotic and hormonal stress groups in *Arabidopsis*.

Gene Name	Identifier	FC^a^	*P*-value	Stress^b^	Source
***BUG*s**					
*CORI3*	*At4g23600*	*24.8*	*0.1640*	B;A;H	^10^
*NATA1*	*At2g39030*	*74.6*	*0.3209*	B;A	This study
*CCR2/GRP7*	*At2g21660*	*32.6*	*0.2516*	B;H	^68,69^
*RBCX1*	*At4g04330*	*39.9*	*0.3405*	B;H	This study
*At1g56300*	*At1g56300*	*26.7*	*0.1913*	B;H	This study
*At3g44860*	*At3g44860*	*23.3*	*0.1466*	B;A	This study
*At3g51660*	*At3g51660*	*17.8*	*0.1589*	B;H	This study
*JAZ1*	*At1g19180*	*15.9*	*0.2099*	B;H	This study
*KIN2*	*At5g15970*	*15.1*	*0.3312*	A	^69,70^
*SRG1*	*At1g17020*	*52.7*	*0.5086*	A	^11^
*ELI3-2*	*At4g37990*	*75.2*	*0.4841*	A	^10^
*PR1*	*At2g14610*	*29.7*	*0.3740*		^69^
*CYP71A13*	*At2g30770*	*83.5*	*0.2205*		^69^
*α-DOX1*	*At3g01420*	*27.9*	*0.5299*		^11,69^
*PDF1.2*	*At5g44420*	*20.1*	*0.0724*		^69^
*VSP2*	*At5g24770*	*2.0*	*0.4478*		^43,69^
***BDG*s**					
*At2g20670*	*At2g20670*	*−4.3*	*0.0060*	B;A;H	^10^
*At1g72060*	*At1g72060*	*−4.2*	*0.1115*	B;A;H	This study
*RAP2.4*	*At1g22190*	−*3.8*	*0.0061*	B;A;H	This study
*DIR1-LIKE*	*At5g48490*	−*13.7*	*0.0003*	B;H	This study
*At1g65490*	*At1g65490*	−*9.7*	*0.0018*	B;H	This study
*bHLH*	*At5g50915*	−*9.4*	*0.0186*	B;H	This study
*HAD*	*At2g41250*	−*7.9*	*0.0006*	B;H	This study
*MLO12*	*At2g39200*	−*4.6*	*0.4050*	A	^26^
*CER3*	*At5g57800*	−*2.9*	*0.0078*		^68,69^

^a^Fold change (FC) was calculated by dividing the expression of *B. cinerea*-infected by that of non-infected samples. A twofold difference in expression level between *B. cinerea*-infected and non-infected samples was set for considering a gene to be *B. cinerea* up-/down-regulated genes (*BUG*s/*BDG*s), which were obtained from Dataset S1 (Supporting Information).

^b^Data of up- and down-regulated genes in response to stress groups were obtained from Dataset S8 (Supporting Information).

-, down-regulation; B, biotic stress; A, abiotic stress; H, hormonal stress.

The DEGs common to all stresses were further investigated. For example, up-regulated gene, *coronatine induced 3/JA responsive 2* (*CORI3*/*JR2*; *At4g23600*), was common in response to all stresses (Table 2; Dataset S8, Supporting Information). The three altered expressed genes by the 11 tested stresses, *RAP2.4* (*At1g22190*), *At1g72060* and *At2g20670*, were found to be commonly down-regulated. Overall, our data emphasize on the complex nature of multiple stress responses and support the importance of studying plant stresses in combination.

The GO annotation was established on commonalities of *Arabidopsis* upon inoculation with *B. cinerea* and other pathogens, exposure to abiotic and hormone challenges. Based on the functional similarities of their encoded proteins, *BUG*s or *BDG*s were grouped with those up- or down-regulated belonging to other stress classes. According to AGI locus identifiers, 45 functional categories were classified into three major categories upon their biological processes, molecular functions and cellular components (Fig. S4, Supporting Information). Our analysis revealed that the general “response to stresses” was the category of most up-regulated clustered genes when plants were inoculated with *B. cinerea* and abiotic stress-affected group. The dominant subcategory ‘signal transduction’ was highly associated with plant defense against pathogens and abiotic cues. The ABA insensitive 1 (ABI1)^31^ was up-regulated by *B. cinerea* and bacterial pathogens (Dataset S5, Supporting Information) as well as the SA and ACC hormones (Dataset S7, Supporting Information). This suggests that plant hormones are tightly associated with defense against *B. cinerea* and other environmental stresses. Enzymatic activities including kinases were also among the dominant subcategories in *BUG*s and other groups (Fig. S4, Supporting Information). In addition, “cell wall” term within the cellular component was highly up-regulated in the tested groups; and the wall-associated kinase 1 (WAK1)^11^ was also induced by multiple stresses (Datasets S5 and S7, Supporting Information).

Different GO terms of *BDG*s encoding for structural activity, receptor binding/activity or enzymatic activity proteins were highly down-regulated in response to biotic, abiotic or hormonal stresses, respectively (Fig. S4, Supporting Information). The “electron transport/energy pathways” was the category that most down-regulated genes belonged in response to *B. cinerea*/biotic stress group and *B. cinerea*/hormonal stress group; whereas, “plastids” and “ribosomes” were the dominant subcategories in the cellular component. Consistent with previous findings, our data suggest rapid metabolic repression of photosynthetic proteins when plants are under stress including infection with *B. cinerea* (Fig. S4, Supporting Information)^10^. Many reports have shown that *B. cinerea* induces/represses several genes particularly those encoding developmental, structural and regulatory proteins in *Arabidopsis*^5,10,32^. Our data suggest connections between gene expression alternation and mechanisms underlying stress resistance/tolerance during multiple stress exposure.

**Figure 3:**
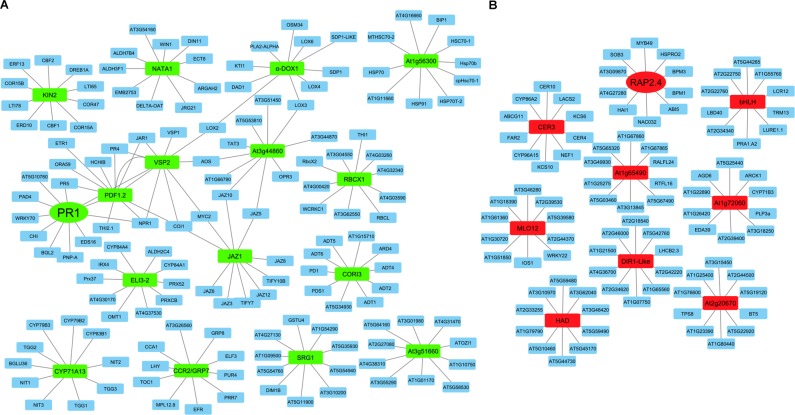
Protein-protein interaction (PPI) network analyses of selected differentially expressed genes (DEGs). Top (**A**) *Botrytis cinerea* up-regulated genes (*BUG*s; green rectangular node); and (**B**) *B. cinerea* down-regulated genes (*BDG*s; red rectangular node), and their neighboring nodes/proteins (blue) are shown. Grey lines represent direct connections/interactions between DEGs (*BUG*s and *BDG*s) and the interacting nodes/proteins, or nodes/proteins with each other. Related to AP2.4 (RAP2.4; red oval node) proteins were further analyzed *in vivo*.

Our knowledge of biological mechanisms related to *B. cinerea* infection is still meager^17,22^. Therefore, we constructed interactome networks of commonly regulated genes *in silico*. *Arabidopsis* PPI network of DEGs after exposure to groups that belong to multiple environmental challenges and hormonal treatments were annotated by calculating their interactive degrees obtained from the STRING database^33^. The interactive networks displayed clique in the sub-network, suggesting that the hub proteins may form a super complex to play important roles in stress response. As a result, a total of 258 nodes were demonstrated to be involved in network construction and 250 edges were established in the network. Key DEGs, such as those encoding RAP2.4 protein possessed degrees of 10 were markedly more compared with those of other proteins (Fig. 3; Dataset S9, Supporting Information); and functional analysis of *RAP2.4* was further studied. Together, this indicates that the dynamic interactive networks between commonly regulated genes may help decide whether certain network topologies can explain experimental observations.

### qRT-PCR validation for the microarray results

**Figure 4:**
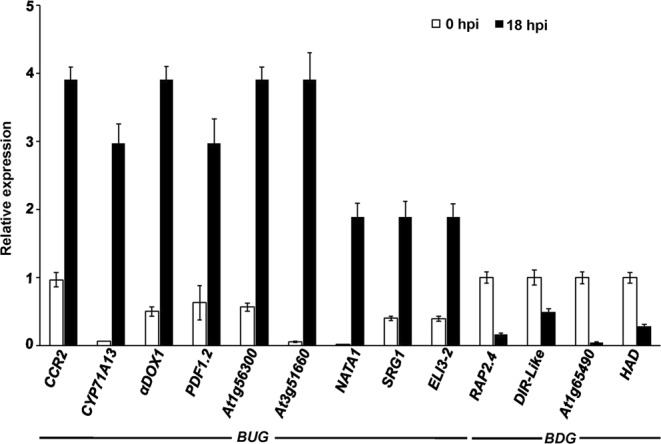
Expression levels of *Botrytis cinerea*- and other stress-regulated genes in response to *B. cinerea* treatment. Relative expression levels obtained by qRT-PCR for selected top 15 common up- and down-regulated genes across all stresses in response to *B. cinerea* infection at 18 hpi (Table 2). Gene expression in *Arabidopsis* wild-type plants inoculated with *B. cinerea* at 18 hpi were compared to non-inoculated samples (0 hpi) and normalized to the expression of the control gene, *Arabidopsis Actin2* (*AtActin2*). Error bars for qRT-PCR values are standard errors (*n* = 3). hpi, hours post inoculation.

To confirm the changes in gene expression revealed by the microarray analyses, 13 DEGs were selected based on the microarray data for verification tests (Fig. 4). We performed qRT-PCR on *Arabidopsis* leaves infected with *B. cinerea* at 0 and 18 hpi. The transcript levels of the DEGs (9 up-regulated; 4 down-regulated) with altered expression in response to various stress treatments (Table 2), were quantified and compared with the obtained microarray analyses. Although the FC values observed in the two expression methods differed somewhat, the tested DEGs displayed comparable patterns in transcript accumulation in the analyses of the two approaches (Table 3; Fig. 4). In response to *B. cinerea*. For example, the transcript levels of the nine *BUG*s (*CCR2, CYP71A13, α-DOX1, PDF1.2, At1g754300, At3g51660, NATA1, SRG1* and *ELI3–2*) were increased (Fig. 4), similar to that of the same set of genes identified in the microarray analyses. On the other hand, *RAP2.4*, *DIR-Like*, *At1g5490* and *HAD*, which were considered as *BDG*s, were also repressed by *B. cinerea* at 18 hpi. In general, a similar trend of expression was found in both the microarrays and the qRT-PCR.

**Table 3:** Details about the microarray treatments that were obtained from the Botany Array Resource (BAR) and used for data analysis in this study.

Type of stress	Treatment	NASCArray reference	n
Biotic stress	*Botrytis cinerea*	NASCArray-167	2
	*Pseudomonas syringae* pv. *DC3000*	NASCArray-120	3
	*P. syringae* pv. *avrRpm1*		3
	Alternria brassicola	NASCArray-330	2
	*Peirs rapae*		2
Abiotic stress	Oxidative stress	NASCArray-143	6
	Wounding	NASCArray-145	6
Hormonal stress	Salicylic acid	NASCArray-192	1
	Methyl-jasmonate	NASCArray-174	3
	1-aminoacyclopropane-1-carboxylate	NASCArray-172	3
	Abscisic acid	NASCArray-176	3

### Mutations in *RAP2.4* enhanced resistance to *B. cinerea*

**Figure 5:**
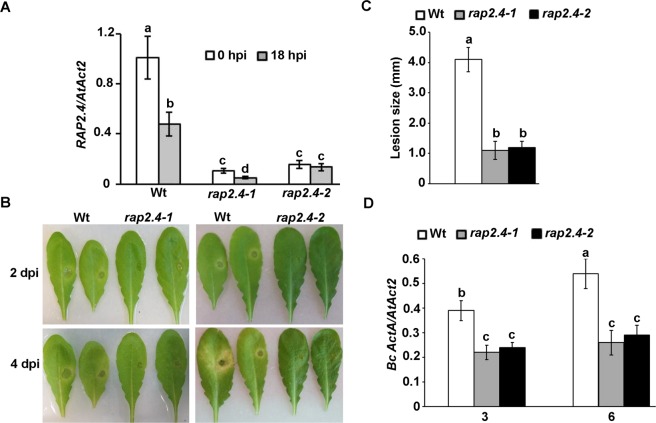
Reduced *RAP2.4* transcript levels enhanced resistance to *Botrytis cinerea*. (**A**) Relative expression using qRT-PCR; (**B**) disease symptoms; (**C**) lesion diameter; and (**D**) fungal growth in leaves of *rap2.4* T-DNA insertion mutant plants after drop-inoculation with *B*. *cinerea*. In (**A**), gene expression in *Arabidopsis* wild-type, *rap2.4-1* and *rap2.4-2* plants inoculated with *B. cinerea* at 18 hpi were compared to non-inoculated samples (0 hpi) and normalized to the expression of the control gene (*AtActin2*). In (**C**,**D**) data represent mean values ± SE (*n* = 20). ANOVA and Duncan’s Multiple Range Test were performed to determine the statistical significance between the mean values using SAS software^67^. Mean values followed by a different letter are significantly different from each other (*P* < 0.05). All assays were repeated independently in triplicates with similar results. Wt, Col-0 wild-type; *RAP2.4*, *related to AP2.4* gene; *Bc ActA*, *B*. *cinerea ActinA* gene; *AtAct2*, *Arabidopsis Actin2* gene; dpi/hpi, days/hours post-inoculation.

From the microarray data, *RAP2.4* was selected on the basis of its expression profiles for further analysis. Two mutant alleles in *RAP2.4* gene displayed very low basal and repressed *RAP2.4* expression compared to wild-type plants (Fig. 5A). The *rap2.4-1* showed the lowest transcript levels of *RAP2.4* at 18 hpi with *B. cinerea*; and therefore, this T-DNA insertion line was used in further experiments. We assumed that *RAP2.4* more likely plays a role in defense. Two homozygous T-DNA insertion lines were inoculated with the fungal pathogen *B. cinerea*, evaluated for disease resistance and compared with inoculated *Arabidopsis* wild-type (Col-0) plants –a relatively resistant ecotype.

After inoculations, disease lesions remained restricted in both T-DNA insertion mutant lines of *RAP2.4* and exhibited a more resistant phenotype than the wild-type disease phenotype at all time points of infection (Fig. 5B). In wild-type plants, lesions expanded until 4 days post inoculation (dpi), with chlorosis surrounding them. Obviously, when we measured the diameter of the lesions, the *rap2.4-1* and *rap2.4-2* mutants demonstrated smaller lesions than the wild-type plants (Fig. 5C), confirming that in the observed disease phenotype. To measure disease development more precisely, fungal biomass accumulation of *B*. *cinerea ActinA* (*Bc ActinA*) per leaf was measured at 3 and 6 dpi for each of the mutant lines. Compared to growth in wild-type plants, there was a clear decrease in the growth of fungal biomass in the *rap2.4* mutants (Fig. 5D). This suggests that *RAP2.4* gene contributes to plant immunity toward *B. cinerea*.

### Down-regulation of *RAP2.4* alters the expression of defense-regulated genes in response to *B. cinerea*

**Figure 6:**
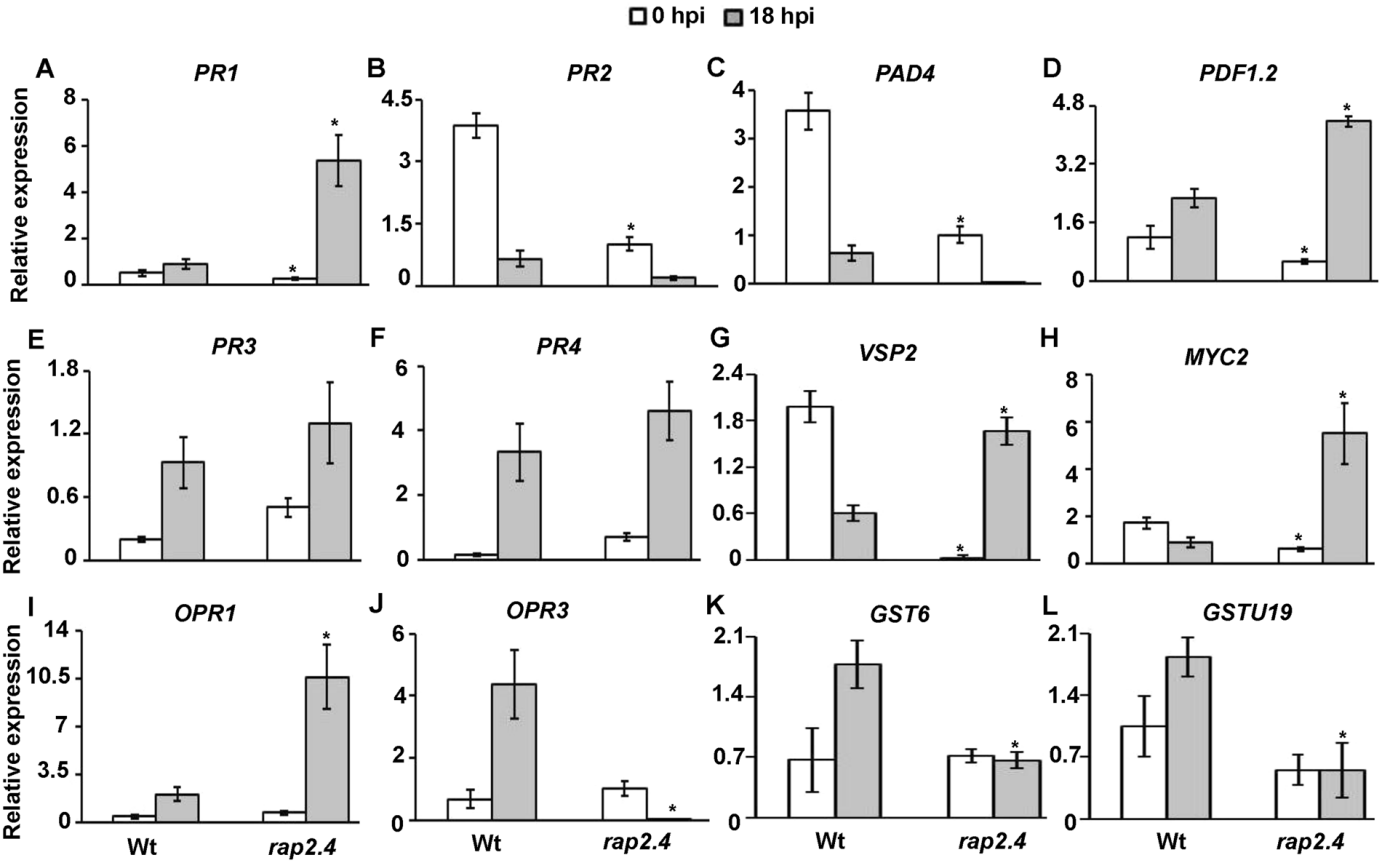
Expression of defense response-related genes during *B. cinerea* infection. Relative expression using qRT-PCR of selected (**A**–**C**) salicylic acid (SA); (**D**–**F**) jasmonic acid/ethylene (JA/ET); (**G**–**J**) JA and/or 12-oxo-phytodienoic acid (OPDA); and (**K**,**L**) cyclopentenone pathway-associated genes. Gene expression in *Arabidopsis* wild-type (Wt) and *rap2.4*-*1* plants inoculated with *B. cinerea* at 18 hours post-inoculation (hpi) were compared to non-inoculated samples (0 hpi) and normalized to the expression of the control gene (*AtActin2*). Error bars for qRT-PCR values are standard errors (*n* = 3). Mean values followed by an asterisk are significantly different from the corresponding wild-type control at the specified time of inoculation (*P* < 0.05). All assays were repeated independently in triplicates with similar results.

In order to link *RAP2.4* function in defense to specific pathway(s), we assessed the response of *B. cinerea*-infected tissues to molecular markers of different signaling pathways. The transcript levels of the SA-mediated defense-associated genes, *PR1*, *β-1*,*3-glucanase* (*BGL2*/*PR2)* and *phytoalexin deficient 4* (*PAD4*)^34^ were determined in *rap2.4-1* mutant. The basal expression levels of all SA-associated genes in uninfected plants revealed significant reduction in *rap2.4* compared to wild type (Fig. 6A–C). Although the transcript of the SA marker gene, *PR-1*, increased at 18 hpi with *B. cinerea* in wild-type plants, the expression was significantly higher in *rap2.4* (Fig. 6A). This suggests that *rap2.4* plants respond faster in terms of *PR-1* expression; thus, the increased *PR-1* levels may be an indirect consequence of the decreased rate of fungal growth in *rap2.4* plants. After *B. cinerea* inoculation, *PR2* and *PAD4* showed a similar pattern of expression between *rap2.4* and wild type plants infected with *B. cinerea* although the repression was much lower in the mutant plants (Fig. 6B,C). This suggests that the basal expression level of SA-related defense genes might be dependent on *RAP2.4*.

At 18 hpi with *B. cinerea*, the transcript levels of JA/ET pathway defense marker genes, *PDF1.2*, *PR3* (*β-chitinase*) and *PR4* (*hevein-like*) were significantly induced in both wild-type and *rap2.4* mutant plants (Fig. 6D–F). The increase in the expression of *PR3* and *PR4*, but not *PDF1.2*, was comparable in wild-type and *rap2.4* plants. Hence, these *B. cinerea*-induced genes which are activated by the oxylipins, JA and/or OPDA, are COI1-dependent^35,36^ and are negatively regulated by the TF, MYC2^37^. In wild-type plants, there was repression in *VSP2* in response to *B. cinerea*; thus, the transcript levels of *VSP2* were markedly increased in *rap2.4* after inoculation (Fig. 6G). Similar to *VSP2*, healthy *rap2.4* plants expressed lower basal levels of *MYC2*, but the transcript accumulated to high levels within 18 hpi (Fig. 6H). This is in contrast to the suppression of *MYC2* in *B. cinerea*-inoculated wild-type plants. This suggests that the down-regulation of *RAP2.4* may require oxylipins during pathogen infection.

There was an increase in transcript levels of *12-oxophytodienoate reductase 1* (*OPR1*) when wild-type and *rap2.4* plants were challenged with the necrotrophic fungal pathogen *B. cinerea* (Fig. 6I). The induction was significantly increased in *rap2.4* mutant. On the other hand, the expression of *OPR3* was altered in *rap2.4* mutant plants after infection (Fig. 6J). The expression of genes that were responsive to the cyclopentenones, phytoprostanes (PPs) or 12-oxo-phytodienoic acid (OPDA)^38,39^ was also analyzed. The *Arabidopsis* detoxification-related genes, *GST6* and *GSTU19*, were up-regulated in in wild-type-infected plants (Fig. 6K,L). No induction of the same genes was observed in *rap2.4* mutant after infection, indicating that there is a common regulation between electrophilic oxylipins and *B. cinerea*, and that *RAP2.4* plays a major role in this regulation. Overall, these results suggest that the down-regulation of *RAP2.4* gene alters the expression of JA- and/or cyclopentenone-mediated response genes, which may result in the increased resistance of *rap2.4* mutants to the necrotrophic pathogen *B. cinerea*.

## Discussion

Plants are often affected by diseases mainly caused by pathogens^10,40^ with a consequent serious reduction in plant growth, development and productivity. Abiotic stress conditions have a direct impact on plant-microbe interactions, and alternation of plant physiology and defense responses. Plants retain a range of defense mechanisms to combat stress conditions^41^, which involve a variety of metabolic reprogramming events and cellular pathways. Once sensing a single or multiple stress(es), plants show an immediate and evoking response to initiate a complex stress-specific signaling by synthesizing hormones and accumulating phenolic compounds^14^. In this work, we performed a meta-analysis of transcriptomic data related to environmental challenges in *Arabidopsis* publicly available at present to explore the complexity of the transcriptional changes of *Arabidopsis*. For that reason, we analyzed the transcriptome of *Arabidopsis* at one time point after pathogen infection, insect infestation or abiotic stress using Affymetrix ATH1 whole-genome GeneChips. We aimed to determine which genes, pathways, gene set categories and predicted PPI networks may play a key role in specific responses to environmental stresses (pathogen infections and abiotic cues). Our focus in the current study was not only on plant responses to the necrotrophic pathogen *B. cinerea*, but also to the virulent and avirulent bacterial strains of *P. syringae* pv. *tomato*, the other fungal pathogen *A. brassicicola* and the herbivore insect *P. rapae*. We also extended our analysis to include oxidative stress and wounding on *Arabidopsis*. Because alterations in the level of phytohormones have some prominent responses against environmental stresses *in planta*, we studied the effect of SA, MeJA, ACC and ABA treatments.

There is an increasing demand for more meta-analysis of transcriptomic studies^7,41^, which can be featured for many reasons. First, the transcript levels are highly affected by changing environmental conditions. Second, the inconsistency in the generated results, coming from field studies, can be affected by other disturbing factors. Third, the integration of high-throughput technologies (transcriptomics, proteomics and metabolomics) is of a concern nowadays due to the high costs and qualified experts in “omic” analyses. Moreover, the commonalities of independent studies will identify genes strongly associated with the studied stresses in order to focus on the functional analysis of these common DEGs^7,42^. Here, the well-defined analysis pipelines, standardized approaches for data analysis in addition to the availability of datasets to public were among the reasons that microarrays were selected over any other transcriptomic approaches.

In *Arabidopsis*, we found 6.8% and 5.3% of the transcriptome was considered up- and down-regulated in response to *B. cinerea*, respectively, which is in agreement with previous data in a number of studies^5,10,26^. The time point for global expression profiling was set to 18 hpi with *B. cinerea*, due to the fact that most changes in gene expression occur between 18 and 30 hpi^5,10,11,43^. In addition, the findings of our microarray meta-analyses on *Arabidopsis* plants individually treated with a tested single biotic stress, abiotic stress or hormone, are comparable to those in the literature^5,10,18,44,45^. It must be noted that in all *Arabidopsis*-stress combinations, many genes showed a more than twofold change in expression at the time point tested. Although several studies have reported meta-transcriptomic analysis combining different environmental stresses, this report linked *B. cinerea*-infected plants with other biotic and abiotic stresses at the transcriptional level. More than 50% of *BUG*s were also induced by any of the *P. syringae* pv. *tomato* strains. In addition, 40–50% of all consistent changes elicited by *A. brassicicola* and *P. rapae* were consistently triggered by *B. cinerea*. This suggests that these genes are commonly activated or repressed during these *Arabidopsis*-attacker interactions.

On the other hand, the number of co-regulated genes in response to *B. cinerea* and the tested *Arabidopsis*-abiotic challenge combinations was much lower. We also investigated the role of hormones in the regulation of the overlapping gene sets of *Arabidopsis*-*B. cinerea* interaction. We identified probesets representing individual hormone-responsive genes among the selected *B. cinerea*-responsive genes. Comparison of the hormone-responsive genes with that of *B. cinerea*-responsive probesets revealed that 34%, 33%, 29% and 36% of the *BUG*s are responsive to SA-, JA-, ACC- and ABA, respectively (Table 1). The percentages of all hormonal responsive genes with the *B. cinerea*-repressed changes were even higher, indicating that hormones play a dominant role in the transcriptional reprogramming of *Arabidopsis* in response to *B. cinerea* infection. This is confirmed by another study of which the expression of *BUG*s is also affected by *ethylene-insensitive 2* (*ein2*), *coronatine insensitive 1* (*coi1*) and SA-deficient (*nahG*) mutations^5^, suggesting a regulatory role of hormones in mediating gene expression by *B. cinerea* which may have effects on disease responses. It has been reported that a number of *P. syringae*-, *A. brassicicola*-, and *P. rapae*-induced genes are also considered as JA-responsive genes^44^. Although pathogen/insect attackers with very different modes of action (e.g., *B. cinerea*, *A. brassicicola*, *P. syringae* and *P. rapae*) may induce similar sets of responsive genes, the majority of these genes are affected by a specific attacker. This suggests that hormones and other factors may enable fine-tuning the transcriptional machinery of defense response.

Interestingly, the eleven stress treatments induced only one gene and repressed three genes that could be considered as common regulators of the overlapping gene sets. The comparative microarray data analysis demonstrated that *CORI3*/*JR2* was commonly up-regulated and *RAP2.4*, *At1g72060* and *At2g20670* were found to be commonly down-regulated (Fig. S3, Supporting Information). The *CORI3/JR2* gene encoding cystine lyase is an enzyme that generates an ET precursor, and has been previously reported to be induced by *B. cinerea*, cold, drought and oxidative stresses^10^, *P. syringae*^40^, wounding^46^ and MeJA^47^. *Arabidopsis* RAP2.4d, a common repressed gene to all stresses in test, is a member of RAP2.4 (AP2/DREB-type TF) family, was down-regulated in response to cold, light and ET but up-regulated in response to oxidative stress, increased salt and drought^48,49^, suggesting a role of RAP2.4 to coordinately regulate multiple abiotic stress responses. In addition to *B. cinerea*, the transcript levels of *At1g72060* was reduced by cold, drought and oxidative stress^10,32^; and *At2g20670* was repressed by heat, salinity and osmotic stress^11^.

To get an insight into the function of commonalities of *B. cinerea* and other stress groups, we categorized the biological function according to the GO tool. Some of these functional categories covered a relatively large proportion of the *Arabidopsis* genome e.g. “electron transfer/energy pathways”, representing most of the annotated genes commonly regulated by *B. cinerea*-biotic stresses and *B. cinerea*-hormonal stresses, but not *B. cinerea*-abiotic stress group. Thus, most of *B. cinerea*-abiotic stress group belonged to the functional category “biological processes” and “response to stress/stimulus”. The number of up-regulated genes predicted to be involved in “response to biotic and abiotic stress” in all *Arabidopsis* biotic stress combinations tested was similar to observations done by the four biotic stresses of *P. syringae*, *A. brassicicola*, *P. rapae* and the feeding thrips (*Frankliniella occidentalis*)^44^. This distribution of the identified DEG sets over the various functional classes within the genome makes it possible to better understand the importance of the functional category in plant response. Analysis of GO data revealed that several *B. cinerea*- and abiotic stress-induced genes were in favor in “receptor binding” and “TF activity”. Another large functional group of DEGs was enriched in the regulation of cellular components. These categories were consistent with the response of the plant to environmental stress known to derive to strong metabolic readjustment, defense mechanism and turning off the photosynthesis machinery. Our data are in accordance with previous findings in response to biotic and abiotic stresses in *Arabidopsis*^50,51^.

Modern ‘omic’ technologies can generate countless lists of biological identifiers/genes. Visualization of the identified overlapping genes/proteins can provide new insights into phenotypes and better understanding of the biological significance^52^. The co-expression data and PPI networks were incorporated and visualized to identify the regulation of the up- and down-regulated genes/proteins (Table 2). Our analysis of the interactive networks of proteins matches with what has been previously reported that biological networks may elucidate the roles of hub proteins in distinct pathway(s) involved in response to multiple environmental changes^53^. STRING is a distinctive database in a way that it not only allows the visualization of gene/protein lists of an interactome network, but also links it directly to a comprehensive annotation of the gene/protein lists; thereby providing biological implications of these overlapping as well as non-overlapping gene/protein sets^33^. The integration of gene expression data with the interactome network can provide further biological information associated with the function of these genes in response to multiple environmental challenges.

The genes involved in co-expression analysis have been underlying in molecular network formation^54^. The co-expressed genes might be validated by their regulation is such having similar *cis*-regulatory elements for a TF. Thus, co-regulation studies validate the relationship of correlated genes. Among these is RAP2.4, which is involved in ET-activated signaling pathway^49^ and was repressed by *B. cinerea* (Fig. 5). As most RAP2.4 TFs, RAP2.4d protein mediates the fine-control and adjusts the availability of three chloroplast peroxidases^48^. In addition, RAP2.4 can bind to both the dehydration-responsive element (DRE) and the ET-responsive GCC-box^49^. The T-DNA insertion line of *RAP2.4* showed increased resistance to *B. cinerea*. The *B. cinerea*-induced *RAP2.2* which also relies on ET signaling pathway, showed, on the other hand, decreased resistance to this fungal pathogen^55^. This indicates that *RAP2.4* appears to be important in response to abiotic and biotic stresses, particularly in the tolerance to drought^49^ and pathogenesis of *B. cinerea*. Although resistance mediated by SA has been reported to be mainly against biotrophic pathogens, other reports have shown the involvement of SA-mediated genes in response to necrotrophic pathogens including *B. cinerea*^14,22^. Similar to our results, *PAD4* gene was repressed but *PR1* gene was induced after *B. cinerea* infection^22^, regardless of the inoculated genotype (Fig. 6). In comparison to wild-type, *rap2.4* mutant plants exhibited significantly decreased expression levels of *PR1*, *PR2* and *PAD4*, indicating that the basal expression level of SA-responsive genes may depend on *RAP2.4*. In addition, JA/ET-mediated response contributes to plant defense against necrotrophic pathogens^14,22,56^. The JA/ET responsive genes, *PDF1.2*, *PR3* and *PR4*, were induced by *B. cinerea* in wild-type and *rap2.4* plants. The *VSP2* gene that is regulated by the *MYC2* can be activated by JA and wound but suppressed by ET^37^. Our results showed that the expression of *VSP2* and *MYC2* was enhanced in *rap2.4* but repressed in wild type plants after *B. cinerea* infection. These findings are in agreement with a previous study that infections with *B. cinerea* induce *Arabidopsis VSP2* and *MYC2* at 14 hpi, followed by a significant decrease at 24 hpi^28^. This might be attributed the increased levels of the stress hormone ET leading to down-regulation of *VSP2* and *MYC2* in the wild-type-infected plants^57^. Interestingly, our qRT-PCR analysis showed that the reduced transcript levels of *VSP2* (Fig. 6) seems to contradict the results obtained from the microarray data analysis (Table 2). This discordance could be due to the different plant growth conditions and pathogen infection procedures in this study and the datasets obtained from publicly available microarrays.

Previous studies have reported that the cyclopentenone OPDA regulates gene expression in concert with JA to fine-tune the expression of defense genes^16,35^. *Arabidopsis EXLA2* and *OPR3* genes can modulate gene expression through COI1 or the electrophile effect of the cyclopentenones under biotic stress conditions^16,58,59^. In addition, *Arabidopsis* and tomato mutants of *OPR3* enhanced resistance to *B. cinerea*^60,61^. Consistent with that, it was found that the transcript levels of *OPR3* were dramatically reduced in *rap2.4*-infected plants, indicating a common regulation of gene expression in response to electrophilic oxylipins and *B. cinerea*. The detoxification-related genes, *GST6* and *GSTU19*, that are known to be highly up-regulated by OPDA and PPs^38,39^ were also induced in the wild‐type, but not in the *rap2.4* mutant, after infection. This confirms that the regulation of these genes is affected by the down‐regulation of *RAP2.4* toward *B. cinerea*. We speculate that *rap2.4* mutant accumulates JA as well as cyclopentenone oxylipins upon infection with *B. cinerea*. To test this hypothesis, quantification of JA, OPDA and phytoprostane A_1_ (PPA_1_) in *rap2.4* mutant before and after infection is among our priorities. Future research to dissect the importance of RAP2.4 in the regulation of JA- and/or cyclopentenone-mediated gene responses and to map all implicated elements in stress signal transduction pathways should be a major focus.

Our data provided a detailed picture of the highly dynamic interactive network involving several signaling proteins, in which inoculation with *B. cinerea* served as major control parameter to multiple stress responses in *Arabidopsis*. A recent genome-wide association study has revealed the combinatorial effect of *P. rapae* and drought on the regulation of genes in response to *B. cinerea*; thus by differentially regulating the level of resistance to *B. cinerea*^1^. The meta-analysis of the current study revealed that upon individual or multiple stress(es), several common genes play a significant role in the defense mechanism of the plant. We conclude that *RAP2.4* has the potential to serve in plant defense through the regulation of endogenous signal molecules and/or pathogen‐derived effectors. Future investigations into the identification of pathogen‐suppressed *RAP2.4* gene expression and the relationship with membrane‐associated microbe pattern (MAMP)‐triggered defense will help explain the functions of RAP2.4 in innate immunity against *B. cinerea*. The long-term goal is to identify target genes, which can be helpful in breeding programs and agricultural biotechnology, and to generate crop varieties resistant to pathogen challenges, climatic changes and hormonal imbalances.

## Methods

### Plant growth and treatment conditions

We analyzed data from publicly available microarray datasets on *Arabidopsis* plants (ecotype Col-0) either infected or treated with the necrotrophic fungus pathogen *B*. *cinerea*, other biotic (*P. syringae* pv *tomato DC3000* and *avrRpm1*, *A. brassicicola* and *P. rapae*), abiotic (oxidative stress and wounding) or hormonal (SA, MeJA, ACC or ABA) stresses. The microarray datasets were downloaded from NASCArrays at the BAR for *Arabidopsis* functional genomics database (http://bbc.botany.utoronto.ca/)^30^. The reference numbers for the corresponding stresses can be found in Table 3. Briefly, five-week-old *Arabidopsis* plants were inoculated by placing four 5-μl drops of 5 × 10^5^ spore mL^−1^ solution of *B*. *cinerea* on detached leaves. Responses to *B*. *cinerea* infection were assayed at 0 and 18 hpi of adult leaves. For bacterial infections, *P. syringae* pv. *tomato DC3000* or *avrRpm1*, were infiltrated with 1 × 10^8^ CFU mL^−1^. Bioassays using drop-inoculation of 3-µl drops of 1 × 10^6^ spores mL^−1^ of *A. brassicicola* were carried out on detached leaves of 5-week-old plants. Infestations with *P. rapae* were performed by transferring 5 chewing larvae per plant^42^. Responses to these biotic stresses were tested at 0 and 24 hpi.

For the oxidative stress or wounding treatment, 18-day old seedlings were exposed to 10 µM methyl viologen (paraquat) or punctured with pins, respectively, at 0 and 24 hours post treatment (hpt) as previously described^8^. *Arabidopsis* seedlings grown in MS media were treated with 10 µM SA^8^, MeJA, ACC and ABA^62^. Expressions levels of hormonal treatments were determined at 3 hpt. Only shoot tissues were analyzed for all eleven stresses. No infection/no treatment control (designated as 0 hpi/hpt) was used from the obtained dataset NASCArray-137.

### Microarray procedure and normalization method

Affymetrix Expression Console (EC) software was used to treat the publicly available raw CEL files^63^. This included probeset signal integration, background correction and quantile normalization. Transcriptome Analysis Console (TAC) software was used to analyze DEGs.

Affymetrix GeneChip ATH1 genome arrays representing 22,810 *Arabidopsis* genes were considered in each dataset from which, those with the absence (A) and medium (M) signal detection calls were removed and those which showed present (P) were only considered for further analysis. Depending on the treatment, the number of tested samples (*n*) for each replicate varies (Table 3). Three technical replicates were used for the analysis for each gene and their average was taken as the signal intensity. The FC for each gene was calculated by dividing the signal intensity (SI) of that particular gene in the treated data by the SI of that gene in the non-treated^10,11^. The FC gene expression of ≥2 (up-regulated) or ≤0.5 (down-regulated) was set as default filter criteria for significant DEGs at *P* ≤ *0.05*. Log_2_-transformed expression level data were used to generate scatter plots to detect the effect of each stress at 0 and the corresponding 3 hpt for hormones, 18 hpi for *B. cinerea* or 24 hpi/hpt for other biotic and abiotic stresses on plant gene expression. The gene identities and GO across microarray datasets were established using The Arabidopsis Information Resources (TAIR; www.arabidopsis.com). Although we compared gene sets across experiments and identified overlapping patterns of DEGs, the raw data was re-normalized under identical platforms regardless of spatiotemporal variability in the data.

The heatmap was generated using Morpheus (https://software.broadinstitute.org/morpheus/) to visualize large data matrices. To analyze the generated gene expression datasets, the top 50 *BUG*s and *BDG*s were plotted along with their corresponding DEGs of the other tested datasets. Log_4_ values of FC were scaled as −4 to +4; and denoted by color intensity from red (down-regulated genes) to green (up-regulated genes). Black was denoted for the absence of gene expression in the specific dataset. To identify the common DEGs across the datasets, Venn diagrams were generated using Venny 2.1 software (http://bioinfogp.cnb.csic.es/tools/venny/index.html).

### *Arabidopsis* PPI network

The PPI dataset was downloaded from *A. thaliana* Protein Interaction Network (AtPIN)^64^. The interactome data includes the experimentally identified PPIs and computationally predicted interactions. The PPI network was created and visualized using Cytoscape Version-3.7.0^65^. Node-edge attributes for the genes/proteins of interest were downloaded from STRING database (http://string-db.org/)^33^ which contains the known and predicted PPIs of the query. The network was modified based on these genes/proteins using a Cytoscape plugin Style (formerly known as Vizmapper). In the network, nodes represent the proteins/edges; whereas lines represent the interactions/connections. The attributes of the nodes were altered for the visualization of the network in respect to the genes/proteins of interest. Another plugin, BINGO v3.0.3, was used to determine the GO categories, which were statistically overrepresented in the selected set of proteins^66^.

### Fungal culture, plant inoculation and T-DNA insertion lines

For disease assays, *B. cinerea* strain BO5-10 was grown on V8 agar media (36% V8 juice, 0.2% CaCO_3_ and 2% Agar). The fungal cultures were sub-cultured by transferring a piece of agar containing the mycelium to a fresh plate of V8 agar and incubated at 25 °C. In order to study the response of *B. cinerea*, we followed the procedure of disease assays coming from the microarray reference obtained from the BAR. Four rosette leaves from five-week-old soil grown *Arabidopsis* plants were drop-inoculated by placing a 3 µl drop of 2.5 × 10^5^ conidiospores mL^−1^ solution of *B. cinerea* to each leaf^5^. Plants were further kept under a sealed transparent covered plate to maintain high humidity in a growth chamber with fluorescent lighting (186 *μ*E m^−2^ sec^−1^) at 21 °C/18 ± 2 °C day/night temperature and a 16/8 hr light/dark cycle. Responses to *B. cinerea* infection were assayed at 0 and 18 hpi on detached leaves.

Homozygous T-DNA insertion lines of *rap2.4-1* (*SALK_139727*; N677156) and *rap2.4-1* (*SALK_110897*; N667030) were obtained from the NASC (Nottingham, UK). Mutant lines were in Col-0 ecotype background. T-DNA lines were confirmed via qRT-PCR using primers provided in Table S1 (Supporting Information). Fungal biomass was assessed by accumulation of *Bc ActinA* relative to *Arabidopsis Actin2* (*AtActin2*). Lesion size was determined by measuring the diameter of the necrotic area.

### RNA extraction and expression analysis

Samples of *B. cinerea*-inoculated leaves were collected at 0 and 18 hpt for the qRT-PCR analysis. RNA extraction and qRT-PCR expression analyses were performed as described previously^16^. The qRT-PCR amplification was performed using Bio-Rad CFX96 Real-Time PCR System C1000 Thermal Cycler (Bio-Rad, Hercules, California, USA) in triplicates. Reaction mixture (20 μL) contains 100 ng total RNA as a template, 10 μL 2X GoTaq qPCR Master Mix, 0.4 μL 50X GoScript RT Mix for 1-Step qRT-PCR (Promega, Madison, Wisconsin, USA), 0.3 μM each specific left and right primer (Table S1, Supporting Information). The reaction condition was as follows: 40 °C for 15 min for the reverse transcription (RT) followed by 95 °C for 10 min of RT inactivation, 40 cycles of 95 °C for 10 s, 60 °C for 30 s, and 72 °C for 30 s. *AtActin2* was used as an endogenous reference for normalization. Expression levels were calculated by the comparative cycle threshold method, and normalization to the control was performed.

### Statistical analysis

For qRT-PCR assay, three technical replicates of each sample were used with a minimum of three biological replicates. Results were expressed as means ± standard deviation (SD) of the number of experiments. A Student’s *t*-test for the values was performed at *P* < 0.05.

Data of *B*. *cinerea* growth and lesion size in inoculated plants represent the mean ± SD (*n* = 20). Analysis of variance (ANOVA) and Duncan’s multiple range test were performed to determine the statistical significance^67^. Mean values followed by a different letter are significantly different from the corresponding control (*P* < 0.05). All experiments were carried out independently in triplicate with similar results.

## Supplementary information

Supplementary information1

Supplementary information2

Supplementary information3

Supplementary information4

Supplementary information5

Supplementary information6

Supplementary information7

Supplementary information8

Supplementary information9

Supplementary information10

Supplementary information11

Supplementary information12

Supplementary information13

Supplementary information14
