# Supplementary material for: Metatranscriptomic Analysis of Multiple Environmental Stresses Identifies RAP2.4 Gene Associated with Arabidopsis Immunity to Botrytis cinerea
Source: Sci Rep. 2019 Nov 18;9:17010. doi: 10.1038/s41598-019-53694-1 (PMC6861241; doi:10.1038/s41598-019-53694-1)
Supplement: Supplementary file 1 — Supplementary information1 [file 41598_2019_53694_MOESM1_ESM.pdf]

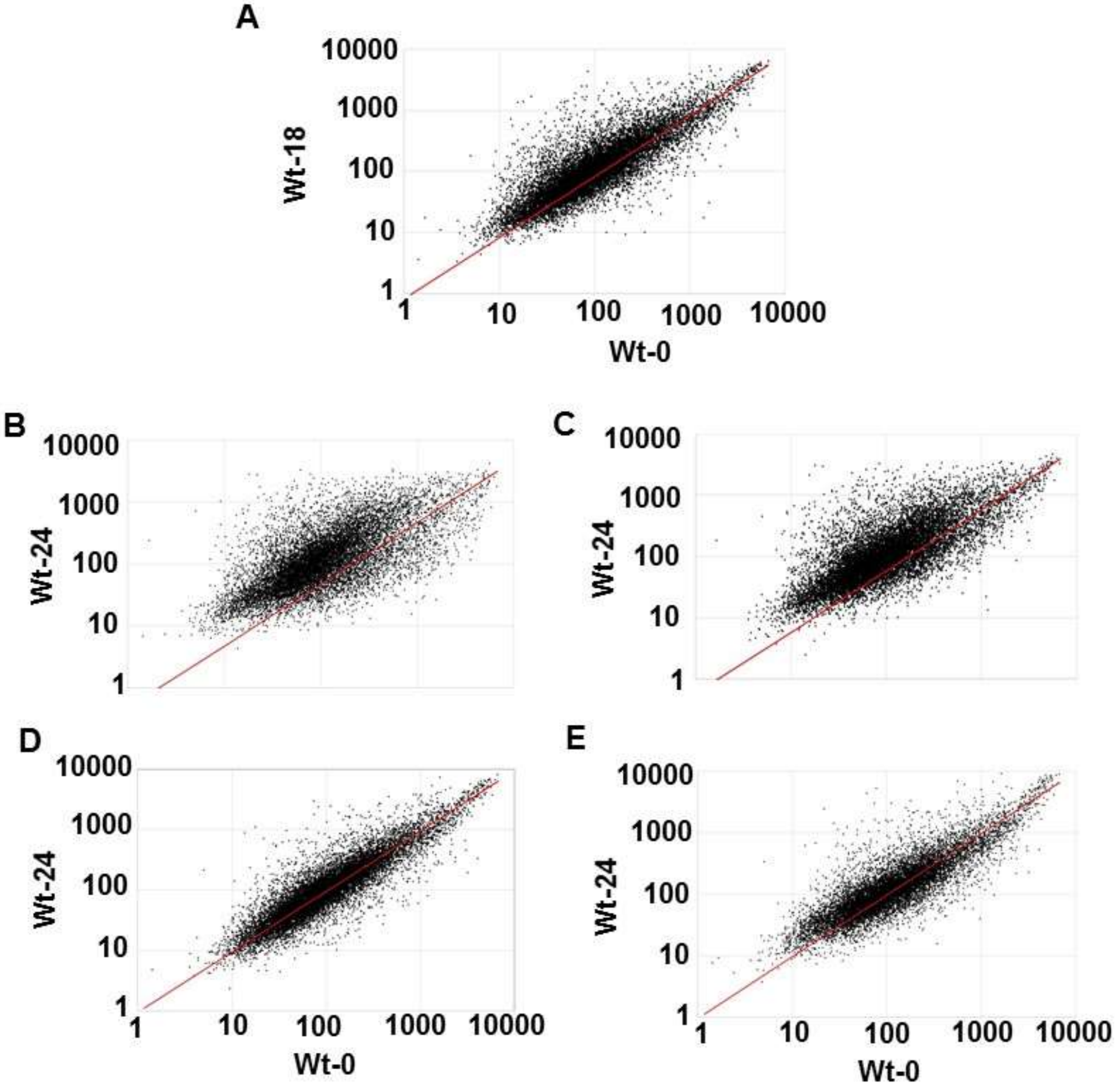

Supplementary Figure S1. Scatter-plot comparison of gene expression of *Arabidopsis* genes treated with different biotic stresses. Normalized expression value for each probe set in untreated wild-type plants at 0 hpi (Wt-0) is plotted on X-axis versus the expression value in wild-type plants after treated with **(A)** *Botrytis cinerea* at 18 hpi (Wt-18); and **(B)** *Pseudomonas syringae* pv *tomato DC3000*, **(C)** *Pseudomonas syringae* pv *tomato avrRpm1*, **(D)** *Alternaria brassicola* and **(E)** *Peris rapae*. At 24 hpi (Wt-24). hpi, hours post inoculation.
