# Supplementary material for: Metatranscriptomic Analysis of Multiple Environmental Stresses Identifies RAP2.4 Gene Associated with Arabidopsis Immunity to Botrytis cinerea
Source: Sci Rep. 2019 Nov 18;9:17010. doi: 10.1038/s41598-019-53694-1 (PMC6861241; doi:10.1038/s41598-019-53694-1)
Supplement: Supplementary file 2 — Supplementary information2 [file 41598_2019_53694_MOESM2_ESM.pdf]

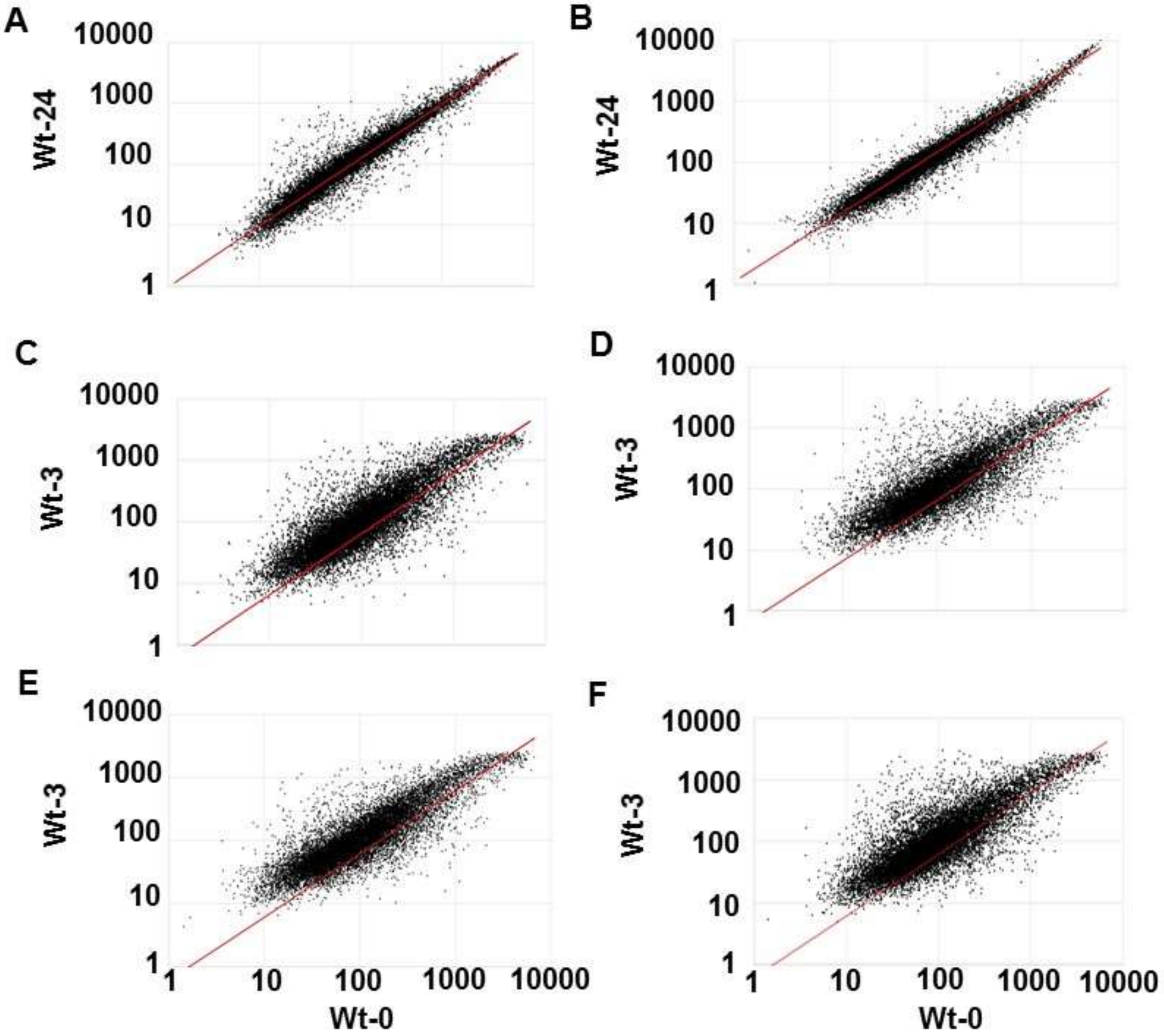

Supplementary Figure S2. Scatter-plot comparison of gene expression of *Arabidopsis* genes treated with abiotic and hormonal stresses. Normalized expression value for each probe set in untreated wild-type plants at 0 hpt (Wt-0) is plotted on X-axis versus the expression value in wild-type plants after treated with **(A)** oxidative stress, and **(B)** wounding, at 24 hpt (Wt-24); and **(C)** salicylic acid (SA), **(D)** methyl-jasmonate (MeJA), **(E)** 1-aminoacyclopropane-1-carboxylate (ACC), and **(F)** abscisic acid (ABA), at 3 hpt (Wt-3). hpt, hours post treatment.
