# Supplementary material for: Metatranscriptomic Analysis of Multiple Environmental Stresses Identifies RAP2.4 Gene Associated with Arabidopsis Immunity to Botrytis cinerea
Source: Sci Rep. 2019 Nov 18;9:17010. doi: 10.1038/s41598-019-53694-1 (PMC6861241; doi:10.1038/s41598-019-53694-1)
Supplement: Supplementary file 3 — Supplementary information3 [file 41598_2019_53694_MOESM3_ESM.pdf]

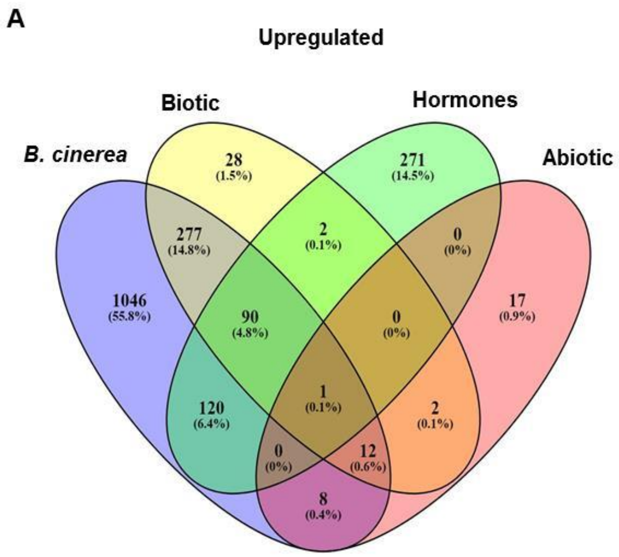

Supplementary Figure S3. Comparison of the number of *BUG*s and *BDG*s in response to stress groups. The Venn diagram showing the number of **(A)** *BUG*s; and **(B)** *BDG*s that were also affected by biotic, abiotic and hormonal stress groups. *BUG*s, *B. cinerea* up-regulated genes; *BDG*s, *B. cinerea* down-regulated genes.
