# Supplementary material for: Metatranscriptomic Analysis of Multiple Environmental Stresses Identifies RAP2.4 Gene Associated with Arabidopsis Immunity to Botrytis cinerea
Source: Sci Rep. 2019 Nov 18;9:17010. doi: 10.1038/s41598-019-53694-1 (PMC6861241; doi:10.1038/s41598-019-53694-1)
Supplement: Supplementary file 4 — Supplementary information4 [file 41598_2019_53694_MOESM4_ESM.pdf]

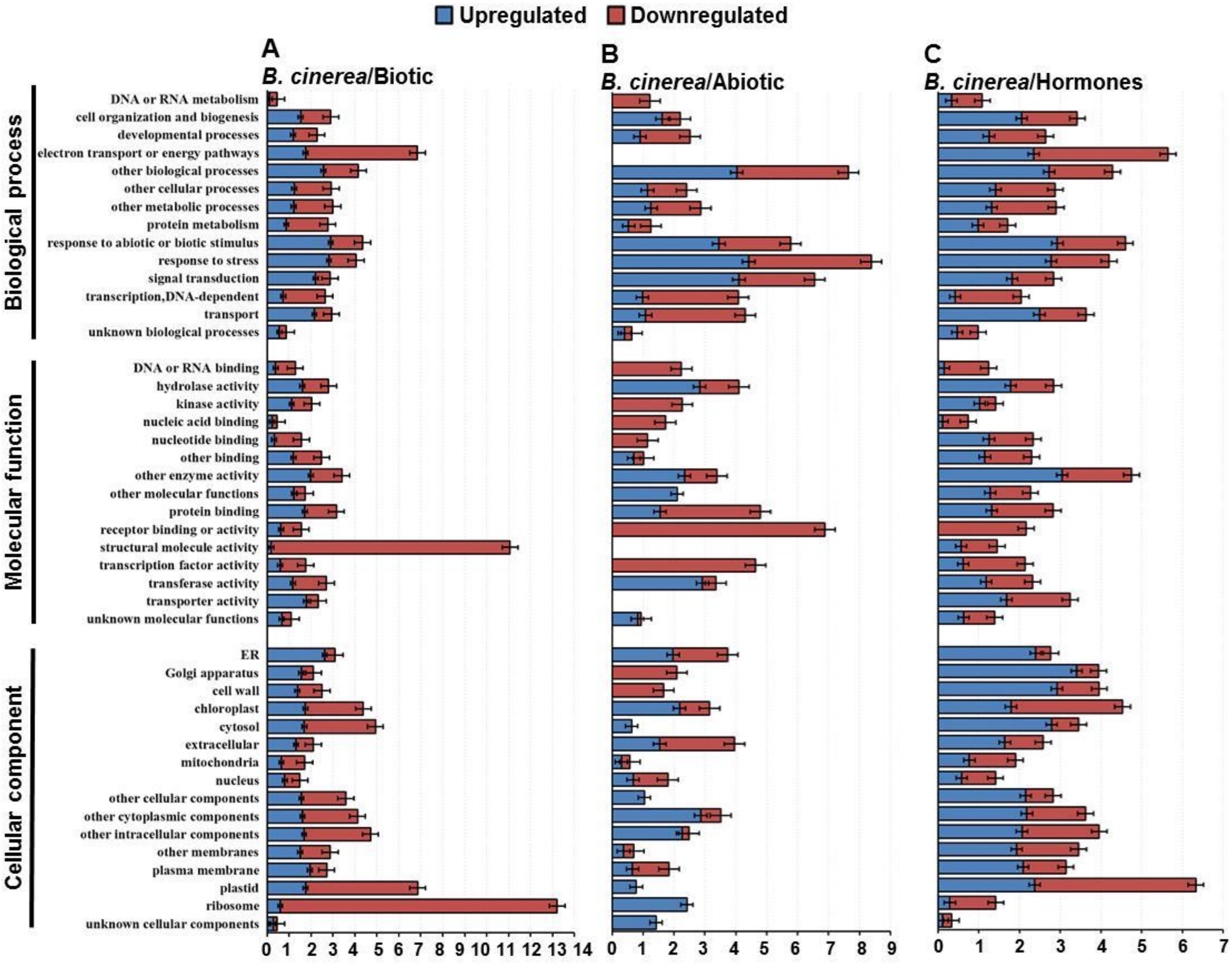

Supplementary Figure S4. GO classification of common DEGs in response to *B. cinerea* and other stress groups. GO of *BUG*s and *BDG*s also affected by biotic **(A)**, abiotic **(B)** and hormonal **(C)** stresses based on biological process, molecular function and cellular components. Gene identifications of 1,554 *BUG*s and 1,206 *BDG*s entered for this analysis. Error bars are SD. GO categories significantly over- or under-represented at $P < 0.05$) are shown. Normalized frequency of genes to the number of genes on the microarray chip was determined as previously described[30].
