# Supplementary material for: Metatranscriptomic Analysis of Multiple Environmental Stresses Identifies RAP2.4 Gene Associated with Arabidopsis Immunity to Botrytis cinerea
Source: Sci Rep. 2019 Nov 18;9:17010. doi: 10.1038/s41598-019-53694-1 (PMC6861241; doi:10.1038/s41598-019-53694-1)
Supplement: Supplementary file 14 — Supplementary information14 [file 41598_2019_53694_MOESM14_ESM.pdf]

Supplementary Table S1. List of qRT-PCR primers (sequence 5' to 3') used in the study.

Gene name	Left primer sequence	Right primer sequence
AtActin2	GTCGTACAACCGGTATTGTGCTG	CCTCTCTCTGTAAGGATCTTCATGAG
RAP2.4	CCACTCATTTTCTCACCATCTTAGT	GTTGTCTCACTCCTCTGTATAGCTTC
BcActinA	ACTCATATGTTGGAGATGAAGCGCA	AATGTTACCATACAAATCCTTACGGA
PR1	CTAACTACAACTACGCTGCGAAC	TTCATTAGTATGGCTTCTCGTTCA
CCR2/GRP7	CACGTAAACCGACTCTAAACCTAGAAA	AGATGTGTAGAACGATGTAGACGAAGT
CYP71A13	GAAGGTAAGAGAAGACGAGGTAAATG	GGTAGAGTTATAAAGAGTTCGCTCAGA
α-DOX1	GATTAGAAGCGGATAGGTTTTTCAC	CATCCTTGAGACTCTCTGTAGTATTCA
PDF1.2	CTTGTTCTCTTTGCTGCTTTCGAC	TTGGCTCCTTCAAGGTTAATGCAC
AT1G56300	AAAAACCATTCCCTCTCTCAACTCT	GAAGTGTTTCTCTGTTCTCAGACGTTA
AT3G51660	GAGATAGTATTTGGAGGGAACAAAG	GTCGCTATGAGTTCTCTCTTAACTTGT
DIR1-Like	CTGACTACACTTGTCTTTGTGGCTA	TAGGTCACACTCTTTAGGGAGACTAGA
AT1G65490	CATCAGTATTTGCTTCTTCCAAGTG	GTACTATGGAGATTGATCAGAAACAGG
NATA1	GTGAATGCTATCAACTTCTATGAGC	AGCTTGTCAATAGCTTGAAGTGCAT
HAD	GTAGGAGATGACCGTAGGAATGATGTA	GAGCAACCTGTTTAAATGACGTAAC
SRG1	ATCAGAAACTAGATTGGGCAGACTT	TATCTCTAAAGGGAAGAGGTAGCTTG
ELI3-2	GGAAGTATGATAGGAGGGATAAAAGAG	CATAATCGGCAGAGATAAGCTCAAT
PR2	AGGAAGGTTCAGGGATGAGTATAAG	TGAAGTAAGGGTAGAGATTCACGAG
PAD4	GGAACAAGCCAAGAAGATACATAGA	GGATATCGAGTAGAGAGTTGCAGAA
PR3	TCATGGGGCTACTGTTTCAAG	TATTGCTCTACCGCATAGACC
PR4	GACCTCGTGGTCAAGCTTCTT	TTGCTACATCCAAATCCAAGC
VSP2	ATGCCAAAGGACTTGCCCTA	CGGGTCGGTCTTCTCTGTTC
MYC2	GAACGAAGATAAAGTTCTA	CAACCGCTCGTAACGCGTAGAA
OPR1	GCACCGCTGAATAAGTACG	GTTAAGTTATGTTGGTCTC
OPR3	GCAGAGTATTATGCTCAAC	GAGGTTTCGGGTACTTCAC
GST6	AGTCAAGAGCCATCACACAGTAC	CTACTGCTTCTGGAGGTCAATAA
GSTU19	ACTAGGACAAGCCATTAAATCCA	GACATTGCGTTGATTGGATTCTAC
